# Genetic aberrations in multiple myeloma characterized by cIg-FISH: a Brazilian context

**DOI:** 10.1590/1414-431X20155034

**Published:** 2016-04-08

**Authors:** P. Segges, E. Braggio, C. Minnicelli, R. Hassan, I.R. Zalcberg, A. Maiolino

**Affiliations:** 1 Centro de Transplante de Medula =ssea, Instituto Nacional de Câncer, Rio de Janeiro, RJ, Brasil; 2 Department of Hematology and Oncology, Mayo Clinic in Arizona, Scottsdale, AZ, USA; 3 Universidade Federal do Rio Grande do Norte, Natal, RN, Brasil; 4 Departamento de Medicina Interna, Serviço de Hematologia, Universidade Federal do Rio de Janeiro, Rio de Janeiro, RJ, Brasil

**Keywords:** Multiple myeloma, Cytogenetic aberrations, cIg-FISH

## Abstract

Genetic abnormalities are critical prognostic factors for patients diagnosed with multiple myeloma (MM). This retrospective, multicenter study aimed to contribute with the genetic and clinical characterization of MM patients in a country with continental dimensions such as Brazil. Genetic abnormalities were assessed by cIg-fluorescent *in situ* hybridization (cIg-FISH) in a series of 152 MM patients (median age 55 years, 58.5% men). Overall, genetic abnormalities were detected in 52.7% (80/152) of patients. A 14q32 rearrangement was detected in 33.5% (n=51), including t(11;14), t(4;14) and t(14;16) in 18.4, 14.1, and 1% of cases, respectively. del(13q) was identified in 42.7% (n=65) of patients, of whom 49.2% (32/65) presented a concomitant 14q32 rearrangement. del(17p) had a frequency of 5.2% (n=8). del(13q) was associated with high plasma cell burden (≥50%, P=0.02), and del(17p) with advanced ISS stages (P=0.05) and extramedullary disease (P=0.03). t(4;14) was associated with advanced Durie-Salmon stages (P=0.008), renal insufficiency (P=0.01) and was more common in patients over 60 years old. This study reports similar frequencies of genetic abnormalities to most series worldwide, whereas the t(14;16) and del(17p), two high risk factors for newly diagnosed patients, exhibited lower frequencies. Our results expand the knowledge on the molecular features of MM in Brazil, a country where innovative therapies that could overcome a poor prognosis for some genetic abnormalities are not always available.

## Introduction

Multiple myeloma (MM) is a malignant condition characterized by the accumulation of clonally proliferating plasma cells (PCs) in bone marrow (BM), and is the second most common hematological neoplasm worldwide (1). Data regarding the incidence of MM in Brazil are currently unavailable and studies on clinical and genetic characteristics of the disease are scarce (2).

Genetic analysis of the malignant PCs have shown several chromosomal abnormalities, which have been considered key factors for the establishment of clonal, malignant populations, detected at very early stages of the disease (3,4). In the last years, high-resolution genomics and transcriptomic approaches [e.g., array comparative genomic hybridization (CGH), single nucleotide polymorphism array CGH, gene expression profiling (GEP), and RNA sequencing] were extensively performed aiming to identify molecular signatures and genetic changes to better discriminate patients with more aggressive disease. Ultimately, the goal is to have validated tools to include in the routine clinical assessment of MM patients to predict clinical outcome (5,6). Fluorescence *in situ* hybridization (FISH) still remains the gold standard test for detecting genomic abnormalities in MM due to its extensive validation by several clinical and research groups (7). Moreover, FISH is a genetic laboratory technique, of which implementation in cytogenetics laboratories worldwide predates other genetic approaches, and has the advantage of allowing a more straightforward standardization of data analysis.

The present study aims to contribute to the molecular characterization of MM by cytoplasmic immunoglobulin (cIg)-FISH in Brazil given the paucity of studies reporting frequencies of genetic abnormalities in this population.

## Material and Methods

BM aspirates from 152 newly diagnosed MM patients were collected from five Brazilian public institutions from 2002 to 2008 and sent for molecular cytogenetic characterization to the Molecular Biology laboratory of the Bone Marrow Transplantation Center (CEMO), Instituto Nacional do Cancer (INCA), Rio de Janeiro, RJ, Brazil. Diagnosis and staging classification of MM followed standard criteria (8 [Bibr B09]).

Main demographic, laboratory (biochemical and hematological tests), and clinical parameters of the patients are shown in Table 1. Briefly, the median age at diagnosis was 55 years, with 9.5 and 12.7% of diagnosed patients being ≤40 and ≥70 years, respectively. The majority of MM patients were men (58.5%) and, at diagnosis, bone lesions were detected in 85.6%, anemia in 33.8%, renal disease in 27.5%, and hypercalcemia in 21.4% of cases. Most patients were in stage III of the Durie-Salmon classification. In addition, 26.4, 40, and 32.6% patients were in stages I, II and III of the International Staging System (ISS), respectively.

**Table:**
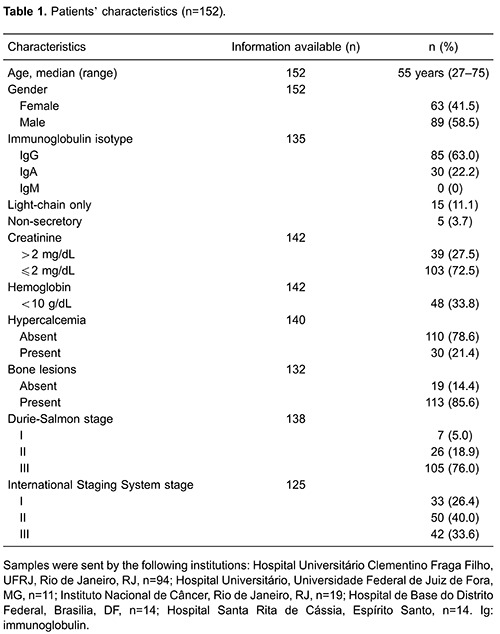

This study was approved by the Ethics Committee of the Instituto Nacional de Câncer (40/04). Written informed consent was obtained from all patients.

Interphase FISH analysis was performed by cIg-FISH as previously described (3). A more detailed description of methods is presented as Supplementary Material. BM samples were screened by cIg-FISH with a panel to detect t(4;14), t(14;16) and del(17p) chromosomal abnormalities, as recommended by the European Myeloma Network (10). The t(11;14) and del(13q) were also analyzed. Sequential analysis of the 14q32 region comprised the use of an IGH break-apart probe as a first assay, followed, when positive, by a second assay to identify the partner chromosome.

Pearson's chi-square and Fisher's exact test were used to analyze associations between dichotomous variables. The Mann-Whitney test was used to analyze associations between dichotomous and continuous variables. Differences were considered to be significant at P<0.05 in two-tailed tests. Statistical analyses were carried out with Statistical Package for the Social Sciences 20.0 (SPSS, IMB, USA) software.

## Results

**Figure 1:**
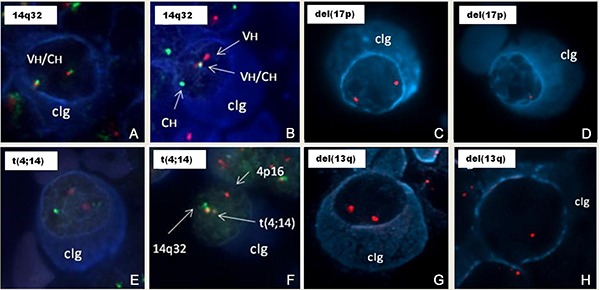
Genetic abnormalities in multiple myeloma. Representative images from the cIg-FISH results. Plasma cells are identified by the blue cytoplasmic stain, which indicates the presence of either cytoplasmic kappa or lambda light chains (cIg). *A* and *B,* analysis with a break-apart probe spanning the IGH locus identifies one pair of juxtaposed red (centromeric) and green (telomeric) signals, and one pair of split red and green signals consistent with an IGH translocation. *C* and *D*, analysis with LSI TP53 probe, normal nuclei and nuclei containing deletion of one copy of the 17p13 regions of a chromosome 17 as indicated by the single red signal. *E*, analysis with a dual fusion probe for t(4;14)(p13;q32) identifies two separate red (MMSET) and green (IGH) signals, indicating absence of the IGH/MMSET translocation. *F*, abnormal malignant plasma cell hybridized with the probe for t(4;14)(p13;q32) shows separate red (MMSET) and green (IGH) signals, and one juxtaposed (fusion) signal, identifying this IGH translocation as t(4;14)(p13;q32). *G* and *H,* analysis with LSI D13S319 probe, normal nuclei and nuclei containing deletion of one copy of the 13q14 regions of a chromosome 13 as indicated by the single red signal.

Genetic aberrations were detected in plasma cells of 52.7% (80/152) of patients. Rearrangements of the 14q32 region were observed in 33.5% of the patients (median percentage of PCs 97%, range 40-100, n=51; Figure 1A and B). The most frequent 14q32-specific rearrangement was the t(11;14), observed in 18.4% of cases, with a median percentage of 57% (range 26-100%) of abnormal PCs. The next most frequent was the 14q32-specific rearrangement t(4;14) (Figure 1E and F), observed in 14.1% of patients (median 86%, range 14-100). The incidence of the t(14;16) was lower, only detected in 1% of patients (median 50%, range 24-89 abnormal PCs). In one remaining case with IGH breaks, the partner chromosome was not identified, likely being another low-incidence gene partner not evaluated in this study. The del(13q) was the most common genetic abnormality and was identified in 42.7% (median 67%, range 22-100, n=65) of patients (Figure 1G and H); in 49.2% (32/65) of cases it was concomitantly found with a 14q32 rearrangement. Eight cases (5.2%) exhibited a del(17p) (Figure 1C and D) and the median proportion of PCs with 17p deletion was 82% (range 18-99%).

Presence of del(13q) was associated with high plasma cell burden (≥50%) (P=0.02); del(17p) was associated with advanced ISS stages (II and III, P=0.05) and extramedullary disease (P=0.03), while t(4;14) was associated with an advanced Durie-Salmon stage (P=0.008), renal insufficiency (P=0.01) and was more common in patients over 60 years old.

## Discussion

MM is currently considered a heterogeneous disease with variable clinical developments and clinical-biological characteristics. Genetic subtypes are associated with unique clinical-pathological features and dissimilar outcomes. Therefore, the European Myeloma Network elucidated the clinical relevance of genetic abnormalities, advising for the detection of t(4;14), t(14;16), and del(17p) by FISH analysis as a standard minimal panel for risk stratification of newly diagnosed MM cases (7).

In the present study, the frequencies of t(11;14), t(4;14) and del(13q) were in agreement with previous studies (3, 11 12 13 14), whereas t(14;16) and del(17p) were observed in lower frequencies than in other series (3,11,15). In a previous study from Brazil (15), the frequencies of t(11;14), del(13q) and del(17p) were similar to the ones herein reported, while a higher frequency of t(4;14) was found in our study (14.1% *vs* 9.3% in the mentioned study) (15). The frequency of t(14;16) has not yet been reported for Brazilian patients. The presence of t(14;16) and del(17p) abnormalities is a high-risk factor associated with poor prognosis (16,17) and the low frequency of these alterations detected in our study compared to international series may be due to the sample size. However, we cannot rule out the hypothesis of under-detection due to the lack of very high-risk patients who were not able to access cancer care facilities and be included in our study sample. A large retrospective epidemiological study of MM in Brazil (2) showed that 70% of patients exhibited advanced disease at the time of diagnosis. Unfortunately, that study did not include the characterization of t(14;16) and del(17p), which could have helped to characterize the existence of molecular risk sub-groups within the clinical high-risk group (2).

In MM, the question whether prognostic risk stratification based on genetic abnormalities retains its value in the context of targeted therapies is a current research issue. For instance, the previous poor prognosis associated with del(13q) and t(4;14) in the context of high-dose therapy and transplantation (11) is no longer effective after the inclusion of novel agents, such as bortezomib and lenalidomide (17,18). In this regard, many centers worldwide have successfully incorporated the combination of immunomodulators and bortezomib in daily practice as induction therapy for patients who are eligible for transplantation. However, those thepapeutic combinations are not available in many other places, due to their high cost. In Brazil, where the majority of patients are treated in the public health system (19), bortezomib is not available as a first-line treatment in all institutions, and lenalidomide has not been approved by the national health regulatory agency (19).^.^ Therefore, in a realistic therapeutic context, the election of genetic targets for risk stratification at diagnosis remains an actual concern, considering the limited availability of novel treatments in a developing country, such as Brazil.

MM risk-based stratification and treatment are being updated continuously. High-resolution genomic assays provide the most comprehensive analysis of genetic abnormalities and are helpful tools for the identification of potential novel markers of disease. GEP analysis has also shown great prognostic power and has been successfully implemented for risk-stratification in MM. However, the high cost of these assays and the complexity of data analysis need to be considered before the definitive incorporation of these technologies in clinical practice. Thus, FISH analysis still remains the standard tool in clinical practice for genomic abnormality detection and disease prognostication, since it has been used for over 30 years and provides results with proven clinical and prognostic value, validated in large cohort studies and with long-term follow-up.

The main limitation of our study is the relatively small sample size and the lack of survival analysis. Although the number of cases was small compared to other large series (European and American), we consider that it is important to know the spectrum of MM genetic alterations in Brazil, where heterogeneous socio-geographic conditions co-exist and an increase in cancer rates (20), as well as the subsequent increase in cancer care investment, is expected.

## Supplementary Material


