# Supplementary material for: Genetic aberrations in multiple myeloma characterized by cIg-FISH: a Brazilian context
Source: Braz J Med Biol Res. 2016 Apr 8;49(5):e5034. doi: 10.1590/1414-431X20155034 (PMC4830026; doi:10.1590/1414-431X20155034)
Supplement: Supplementary file 1 [file 1414-431X-bjmbr-1414-431X20155034-S1.pdf]

Detailed Methodology

Sample preparations

The mononuclear cells from bone marrow aspirates were obtained by density gradient centrifugation at 300 *g* for 30 min at room temperature, using a Ficoll-Hypaque Density Media (Histopaque, Sigma-Aldrich, USA). Cytospin slides were prepared, fixed in 95% ethanol for 5 min, dried at room temperature overnight and stored at –20°C until use.

Fluorescence *in situ* hybridization (FISH)

Interphase FISH analysis was performed by cytoplasmic immunoglobulin (cIg) staining coupled with FISH (cIg-FISH) as previously described (1). Briefly, cytospin slides fixed in alcohol (70%) were stained with either goat anti-human kappa or lambda light chain conjugated with 7-amino-4-methylcoumarin+acetic acid (AMCA) (Vector Labs, USA), followed by incubation with an anti-goat immunoglobulin conjugated with AMCA (Vector Labs) to enhance the intensity of staining. The slides were then denatured in a 37°C 2× standard saline citrate (SSC) for 30 min and dehydrated through a graded series of alcohol concentrations (70%, 85%, and 100%). The probe mix containing 1 µL of probe (10 µg) and hybridization buffer (Vysis, Abbott, USA) was denatured for 5 min at 73°C and placed onto the slides. The slides were denatured at 80°C for 7 min and then hybridized at 37°C for 20 to 24 h on a ThermoBrite system (Vysis, Abbott). Post-hybridization washes (0.4×SSC and 2×SSC/0.3% Tween 20®) were carried out to decrease unspecific hybridization. Only cIg-positive plasma cells (PCs) were scored using a Zeiss Axioplan 2 microscope (Carl Zeiss, Germany) with fluoroisothiocyanate, Texas red, and 4,6-diamidino-2-phenylindole (DAPI) ultraviolet filters (Chromotechnology, USA).

All samples were investigated with the following probes: LSI IGH Dual Color, Break Apart Rearrangement Probe, LSI IGH/CCND1, IGH/FGFR3, IGH/MAF Dual Color, dual fusion translocation probe, LSI TP53 (17p13.1)/CEP 17 probe, and LSI 13 (RB1) 13q14 probe (Abbott).

We scored 100 cells for each probe and recorded the percentage of cells considered abnormal. For each probe, a specific cutoff was determined after having analyzed 200 PCs from 5 normal bone marrow donors. The results were considered abnormal when the percentage of nuclei with abnormal signals exceeded the normal reference ranges, following cutoff levels for positive results according to the European Myeloma Network recommendations (2).

References

1. Fonseca R, Debes-Marun CS, Picken EB, Dewald GW, Bryant SC, Winkler JM, et al. The recurrent IgH translocations are highly associated with nonhyperdiploid variant multiple myeloma. *Blood* 2003; 102: 2562–2567.
2. Ross FM, Avet-Loiseau H, Ameye G, Gutierrez NC, Liebisch P, O'Connor S, et al. Report from the European Myeloma Network on interphase FISH in multiple myeloma and related disorders. *Haematologica* 2012; 97: 1272–1277.
